# Oral Hygiene Profile of Schoolchildren from Bucharest, Romania—How It Can Be Used and Improved for Better Prevention of Oral Diseases

**DOI:** 10.3390/healthcare12131293

**Published:** 2024-06-28

**Authors:** Cristian Funieru, Mihnea Ioan Nicolescu, Cristian Băicuş, Oana Slușanschi, Clara Ilinca Bica, Andreea Moldoveanu, Anca Cristina Perpelea, Adrian Teodor Țandără

**Affiliations:** 1 Division of Preventive Dentistry, Faculty of Dentistry, “Carol Davila” University of Medicine and Pharmacy, 050037 Bucharest, Romania; cristian.funieru@umfcd.ro (C.F.); oana.slusanschi@umfcd.ro (O.S.); ilinca.bica@umfcd.ro (C.I.B.); andreea.moldoveanu@umfcd.ro (A.M.); adrian.tandara@umfcd.ro (A.T.Ț.); 2 Division of Histology, Faculty of Dentistry, “Carol Davila” University of Medicine and Pharmacy, 050474 Bucharest, Romania; 3 Laboratory of Radiobiology, “Victor Babeș” National Institute of Pathology, 050096 Bucharest, Romania; 4 Internal Medicine—Colentina Hospital, Faculty of Medicine, “Carol Davila” University of Medicine and Pharmacy, 020125 Bucharest, Romania; cristian.baicus@umfcd.ro; 5 Division of Organization, Professional Legislation and Management of the Dental Office, Faculty of Dentistry, “Carol Davila” University of Medicine and Pharmacy, 010221 Bucharest, Romania; anca-cristina.perpelea@drd.umfcd.ro

**Keywords:** oral hygiene, students, prevention, diagnosis, management, oral diseases

## Abstract

Background: Oral hygiene is essential for low caries or gingivitis prevalence in children. This study aimed to determine the oral hygiene profile of children from secondary schools (10–17 years) in Bucharest, Romania, and to explore options for preventing their future oral diseases. Methods: The study was cross-sectional, with a sample of n = 1595 pupils. The sample was stratified by clusters in city areas, grades, and the criterion of the presence/absence of in-school dental service. Dental check-ups were performed by using a commune dental examination kit. The oral hygiene simplified index (OHI; Silness and Löe) was used to assess the children’s oral hygiene status. Five questions were used to determine oral hygiene habits. Results: The mean value of the OHI was 1.04. Some differences in oral hygiene scores were found for different socioeconomic variables, such as parents’ education (*p* < 0.05): parents with vs. without a university degree—0.95/0.94 (male/female) vs. 1.06. Most of the children used manual toothbrushes (88%). Conclusions: The analysis of objective data (OHI) revealed a good level of oral hygiene, with many socioeconomic disparities.

## 1. Introduction

Oral health should not be related just to cosmetic dentistry or esthetically pleasing smiles but should be a part of whole-body health [1,2]. It comprises the condition of the teeth, gums, oral mucosa, tongue, masticatory muscles, or alveolars bones, which allow people to chew, swallow, speak, breathe, look good, and be present and comfortable in their social life with self-confidence and without pain [3]. The most common oral diseases are dental caries, gingivitis, and periodontitis. In children, the prevalence of dental caries (% DMFT > 0) is high, ranging from 60 to 90% [4]. Specifically, in Romania, there is a 75% prevalence rate of caries [5]. Plaque-induced gingivitis also has a high prevalence rate in children, 91% [6] or 94% [7], no matter how many gingival areas are counted [Gingival Index Silness and Löe (GI) > 0/0.5]. However, some studies reported low prevalence, but the methodology was different, i.e., using CPITN scores or GI > 1 [8]. Caries and plaque-induced gingivitis are related to dental plaque, and their prevalence and intensity decrease in patients, adults, and children with good oral hygiene profiles.

Some studies provided valid data regarding oral hygiene efficiency in Romanian children. For example, Graça et al. found that Romanian children brush their teeth twice daily less often than children from Portugal and Sweden, and more than half have never used dental floss [9]. Oral health status and preventive behavior are somehow related to the parents’ educational level and impact oral hygiene regularity and efficiency among Romanian children [10,11]. In Bucharest, many schools have dental offices with dentists who work for pupils. They are also important in educating schoolchildren about good oral hygiene [5,6].

Behavior and knowledge of oral hygiene lead to a particular oral hygiene profile. The main goal of this paper is to identify the level of oral hygiene of 10–17-year-old schoolchildren from Bucharest, Romania, by collecting subjective (questionnaire) and objective (oral hygiene score) data and determine how it can be improved to decrease the prevalence of dental caries and periodontal diseases.

## 2. Materials and Methods

### 2.1. Sampling Strategy

The PAROGIM study (where the acronym stands for “Periodontal diseases among gymnasium students” in the Romanian language) was one of the significant cross-sectional studies made in Bucharest, with the primary goals of determining the oral health status and behavior related to oral hygiene of schoolchildren. The materials and methods were already described, along with the previously published data about caries and gingivitis [5,6]. The study included 10–17-year-old children from the 5^th^ to 8^th^ grades, randomly selected by clusters from 59 Bucharest schools (with 56 initially selected and 3 reselected). A total of 85 classes were selected and considered clusters, with an average number of pupils of 25. The classes (clusters) were stratified by grades (5^th^, 6^th^, 7^th^, and 8^th^), city areas (A—central area; B—middle area; C—peripheral area; D—outmost periphery) [12], and the presence or absence of in-school dental services. EPIINFO statistical software for epidemiology, version 3.2.2 (Centers for Disease Control and Prevention, Atlanta, GA, USA), was used to calculate the sample size, which was 1600 for a 58,000 middle-school children population in Bucharest (data from 2010), with a 95% confidence interval, a 2.4% estimation error, and 50% assumed prevalence (for caries/gingivitis).

Thus, an accurate model of the middle-school children population of Bucharest was reproduced on a 2.76% sample size by copying the general characteristics and layers of this population.

### 2.2. Clinical Examinations and Oral Hygiene Assessment

The dental check-ups were performed in schools’ dental offices by using a dental unit and commune dental examination kits (dental mirror, periodontal round-tip probe, and dental tweezer). In the case of schools without dental offices, the check-ups were performed in general medicine offices by using two mobile chairs, one for the examiner and one for the child, and artificial light from a Riester ri-focus LED headlamp (Rudolf Riester GmbH, Tuttlingen, Germany). All the clinical procedures were performed by one experienced examiner (member of the Preventive Dentistry Division) who was trained and calibrated before the study. The calibration process involved 24 children aged 11–14 years who were examined twice, and the results were compared with those of a gold-standard examiner, leading to a Cohen’s kappa score for intra-examiner consistency, which ranged between 0.82 and 0.97 (information about the calibration process was previously published [5]).

Two assessments were used for oral hygiene. First, a Simplified Oral Hygiene Index (OHI-S; by Greene and Vermilion) [13] was used. The buccal surfaces of the first upper molars and right upper and left lower incisors, as well as the oral surfaces of the first lower molars, were examined with a periodontal probe to check for debris and calculus. The OHI-S scores were used to assess oral hygiene status. Secondly, a questionnaire that included five closed-ended questions for assessment of behavior related to oral hygiene (see Table 1) was used next.

### 2.3. Assessment of Economic and Educational Status

The children were asked if their parents had graduated from a university or just from a primary/secondary/high school. The household density measured according to the Mucii score (ratio of number of family members to number of rooms), another instrument for assessing the economic and social status [14]. “A low living standard” was considered when the density score was >1 (more family members than rooms) and “a high living standard” when the score was ≤1 (more rooms than family members or equal).

### 2.4. Data Analysis

All the clinical data and the questionnaire responses were collected on paper and digitalized afterward, using the SPSS processor, version 16 (SPSS Inc., Chicago, IL, USA). Non-parametric tests (Mann–Whitney and Kruskal–Wallis) were performed to identify the differences between OHI-S scores from different categories. Chi-square tests were used for questionnaire analysis and to find the differences among answers. In the case of multiple comparisons, 2 × 3 contingency tables were “broken” into three tables of 2 × 2 each to find out exactly where the differences were.

The study was approved by the Ethics Committee of University of Medicine and Pharmacy “Carol Davila” Bucharest (33788/6 December 2007), Bucharest School Inspectorate and Administration of Hospitals and Medical Services of Bucharest. Every parent or caregiver for all children involved in this study signed the informed consent form.

## 3. Results

In the PAROGIM study, n=1595 pupils were examined, with more than half being girls (52.2%). The schoolchildren were primarily aged between 11 and 14 years (11 years (n = 330; 20.8%); 12 years (n = 397; 25%); 13 years (n = 381; 24%); 14 years (n = 418; 26.4%)), but some of them were 10 (n = 18; 1.1%), 15 (n = 36; 2.3%), 16 (n = 4; 0.3%), or 17 years old (n = 2; 0.1%).

The following numbers of pupils were found in different categories of our sample:

- Grades: 5^th^ (n = 383; 24%), 6^th^ (n = 400; 25.1%), 7^th^ (n = 400; 25.1%), and 8^th^ (n = 412; 25.8%);
- City areas: A—central (n = 458; 28.7%); B—middle (n = 743; 46.6%); C—periphery (n = 318; 19.9%); and outmost periphery (n = 76; 4.8%);
- In-school dental services: yes (n = 882; 55.3%) and no (n = 712; 44.7%);
- Standard of living: low (n = 1045; 65.8%) and high (n = 542; 34.2).

The oral hygiene status was analyzed by using OHI-S variation in different categories (Table 2).

Also, the mean OHI scores for all Bucharest areas were the following: zone A (1.07), zone B (1.02), zone C (1.04), and zone D (0.90).

**Figure 1:**
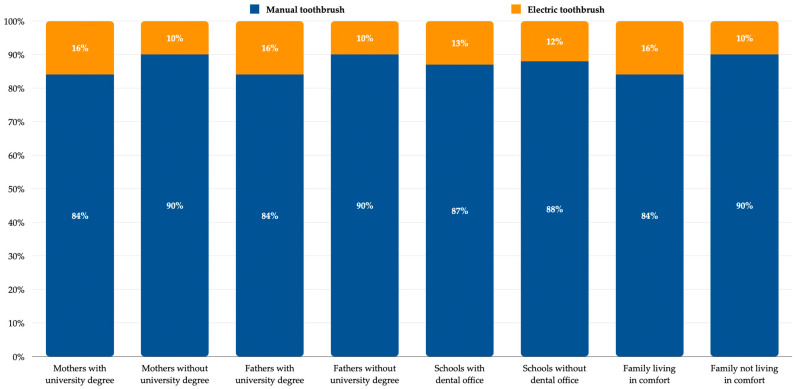
Type of toothbrush used by children.

Oral hygiene behavior was analyzed by using questionnaires. Most of the children involved in this study had and regularly used a manual toothbrush (88%). Using an electric toothbrush is a matter of budget, knowledge, and education (see Figure 1).

Also, the percentages of manual toothbrush use were 87% for zones A and B, 90% for zone C, and 96% for zone D.

Since most schoolchildren used manual toothbrushes, their behavior was studied in relation to toothbrushing, which is part of oral hygiene habits (Table 3).

## 4. Discussion

The OHI-S score analysis showed that the caregivers’ education and socioeconomic status significantly impact children’s oral hygiene. The categories of children whose parents have a university degree or a high living standard undoubtedly have better oral hygiene. However, the link between OHI score and different socioeconomic variables such as income, education, or occupation was also present in other studies [15]. Oral hygiene habits are related to socioeconomic status as a concept and vary across different social classes [16]. The questionnaire analysis showed that children from high socioeconomic categories reported bleeding upon toothbrushing less often. It is well known that bleeding during toothbrushing is due to gingival inflammation, increased brushing force, or both, and gingival inflammation is a consequence of poor oral hygiene. Also, according to our analysis, the smallest percentage of children who changed their toothbrushes in less than two months were those whose parents had not completed a university degree. The result is controversial, but they might not know the proper time for changing the toothbrush, or they might use high toothbrushing force that makes the bristles curved and inactive very soon, which makes them buy another one; perhaps, it is just a chance result.

A questionnaire is a good tool for assessing the oral hygiene habits of different socioeconomic categories. For example, toothbrushing frequency is often associated with socioeconomic variables, such as education or income. Even if the percentages were higher in the case of higher socioeconomic standards, no significant correlations with toothbrushing frequency were found. However, other authors did find such results. Thus, children, adults, or parents with high education or income brush their teeth twice daily more often than those from low social classes [17,18]. In Romania, children and adolescents brush their teeth at least twice daily, which is a strong pattern. At least that is what the studies say, with 74% being found in this study (10–17 years), 67% in Graca et al. (12–18 years) [9], and 49% in rural and 51% in urban areas in Perpelea et al. (7–14 years) [11]. This trend also seems to be maintained among adults (47–58%) [19,20]. It is well known that caries, plaque-induced gingivitis, and periodontal diseases are also associated with poor socioeconomic status [21,22]. Moreover, our previous data showed high prevalence rates of caries and gingivitis, 75% and 91%, respectively, among children from Bucharest, Romania [5,6]. However, this “*strong pattern*” of brushing teeth twice daily does not fit with such a high prevalence of oral pathology. Instead, the children only said and responded “*correctly*” to the questions, rather than having such oral hygiene habits. True or not, it seems like this pattern somehow follows from the parents’ education [10,11].

Using a manual toothbrush (88%) was a more common trend among the studied group of children from Bucharest. Although more children were identified to be using an electric toothbrush when their parents had a university degree or a high living standard compared with their counterparts, the percentages were low, and the differences were not high or significant. However, if so, the trend is somehow correct. In general, an electric toothbrush user seems more educated and has a higher income [23]. Some oral care companies designed powered toothbrush models with special apps that provide real-time feedback on toothbrushing [24]. This tool can be handy these days, because apps can usually be tailored to the patient’s individual needs, and they have also become trendy among children.

Of course, healthcare policies cannot improve people’s socioeconomic status, but oral health programs that focus more on areas with poor socioeconomic standards can be designed. Even though the OHI scores did not reveal any disparities among regions in Bucharest, the prevalence of caries and untreated caries increases from the center to the periphery [5]. So, oral health programs, including oral hygiene education, must focus more on the outskirts or at least concentrate more resources in these areas. Designing oral health programs using mobile apps is a new trend. There are a lot of mobile apps for Android or iOS, such as *MySmile*, *Do I Grid*, *Delta Dental*, *Bad Breath*, *Brush DJ*, etc. [25]. Perhaps an app specially designed for children in Bucharest enrolled in an oral health program might be a solution. The children could connect by using their identification number for the program and follow the instructions provided by the dentists, such as toothbrushing or interdental cleaning. In areas with low socioeconomic standards, the oral health program can provide tablets or other IT devices for using the app.

Pupils who studied in schools with dental services displayed better oral hygiene. Moreover, these children seemed to brush their teeth twice daily and replace the toothbrush every 2–3 months more often than their counterparts. The role of the school dentists is essential in these cases. Better oral health in children who attended schools with dental services in the PAROGIM study (less caries and gingivitis) was already found [5,6]. The role of dental services in schools in decreasing the prevalence of dental caries among children, for example, is already known [26,27]. Today, there are 316 schools and high schools in Bucharest, but only 126 (40%) have in-school dental services [28,29]. This could be a significant problem if the school dental service network does not expand in the next few years and more school dentists do not work with the children. For better oral health and hygiene among children, the dentists must be close to them and provide dental education and services such as prophylaxis, pits and fissure sealing, and dental filling and guide them to an orthodontist or dental surgeon if needed.

The gender difference follows an exciting trend. Our study included an age interval of 10–17 years, but most of our subjects (96%) were between 11 and 14 years old. Firstly, the caries prevalence was determined to be higher in girls. Higher DMFT and FT scores in girls were previously detected [5]. The same results were found in many studies in other countries [30,31,32,33] and Romania [34]. One important cause for this difference might be earlier tooth eruption among girls, a general pattern [5]. The PAROGIM study also found that teeth such as the upper and lower canines, the first and second premolars, and the second molars erupted earlier in girls [35,36]. However, an opposite trend was found for plaque, oral hygiene, and gingival scores. In this paper, girls had a better oral hygiene score and a higher percentage of twice-per-day toothbrushing frequency. Moreover, lower plaque and gingival scores for girls were previously found [6]. Thus, girls seem more interested in brushing their teeth than boys. A higher percentage of girls who reported bleeding on toothbrushing was also detected. This does not match our results so far, because girls also have a low gingival score, but it can fit with the concept that girls reach a maximal gingival inflammation earlier than boys at the beginning of puberty [37]. Moreover, girls brush their teeth more frequently than boys, which implies that they also have more chances for their gums to bleed more often.

Oral health programs for girls and boys cannot be split. It would be unfair and unethical, but different rules and approaches based on what the patterns of the oral pathology for both genders are according to their age can be designed. For example, more focus on oral hygiene in boys or more supervision of caries lesions in girls can be emphasized. Knowing the teeth eruption patterns for boys and girls is also helpful in choosing the proper preventive approaches. In the case of oral health programs based on different apps specially designed for children, the profiles can be gender-customized. For example, one can add cartoon characters, activities, or voices more appropriate for girls or boys.

Our previous results from this study (related to dental caries) suggested that socioeconomic status (measured in our study by the percentage of the parents’ university degrees and standard of living of the families) varies descending from zones A to B, D, and then C [5]. The results related to oral hygiene quality and habits are inconclusive. For example, zone A has the highest percentage of people using a powered toothbrush and a lower rate of bleeding when toothbrushing. Still, it also has the highest OHI score and percentage of toothbrushing time under three minutes.

Our study also has some limitations. For example, being the study cross-sectional, the data could not be followed in time; hence, it could not be observed if they changed somehow over time. Thus, our questionnaire, including the oral hygiene section, was not a standard tool and was not previously used or validated. However, simple questions that were easy for the children to understand were designed to increase the chances of accurate answers.

## 5. Conclusions

The level of oral hygiene measured by using the OHI score was good. In particular, girls and children from different categories, such as those with parents with a university degree, living in comfort, or with direct access to dental services in school, usually displayed better oral hygiene than their counterparts. Analyzing their attitudes and practice, we can say that in some cases, they have even better oral hygiene habits. Designing oral health programs focused on schools and extending the dental office networks in schools may among the solutions for improving children’s oral hygiene status in Bucharest even more, especially based on the expertise and education related to oral hygiene of school dentists.

## Figures and Tables

**Table 1:** The questions that were used to assess the behavior related to oral hygiene.

No.	Question	Possible Responses
1	How often do you brush your teeth?	Twice per day/once per day/less than once per day
2	What kind of toothbrush do you use?	Manual/electric
3	How long do you brush your teeth for?	Less than 3 min/3–5 min/more than 5 min
4	For how long do you use your toothbrush?	Less than 2 months/2–3 months/more than 3 months
5	Do your gums bleed when you brush your teeth?	Yes/No

**Table 2:** Oral hygiene scores (OHI) for every category of children from Bucharest middle schools.

Variable	Schoolchildren Category	OHI (Mean)	OHI = 0 (%)	OHI = 0.1–1.2 (%)	OHI = 1.3–3.0 ^c^ (%)
Gender					
	Boys	1.12 ^a^	4	57	38
	Girls	0.96	5	67	28
Economic and educational level					
University degree	Completed by mothers	0.94 ^a^	5	69	26
	Not completed by mothers	1.06	4	60	35
	Completed by fathers	0.95 ^a^	5	69	25
	Not completed by fathers	1.06	4	60	36
Standard of living	Good (high)	0.98 ^b^	4	66	29
	Poor (low)	1.07	4	60	35
School dental services					
	Schools with dental offices	1.00 ^a^	5	64	30
	Schools without dental offices	1.08	3	60	37
TOTAL		1.04	4	62	33

^a^ *p* < 0.01. ^b^ *p* < 0.05. ^c^ There were only four values of OHI > 3; hence, the corresponding percentages were too low to be shown.

**Table 3:** Manual toothbrushing habits in schoolchildren of Bucharest (grades 5^th^–8^th^)—disparities in gender and socioeconomic categories.

		Manual Toothbrushing ^a^			Length of Toothbrushing (Minutes) ^b^			Time to Toothbrush Replacement (Months) ^c^			Bleeding upon Toothbrushing ^b^	
		Twice/Day or More	Once/Day	Less than One/Day	3<	3–5	>5	2<	2–3	>3	Yes	No
		n (%)			n (%)			n (%)			n (%)	
Schoolchildren category												
Gender												
	Boys	428 (66.1%)	191 (29.5%)	28 (4.4%)	284 (43.9%)	321 (49.5%)	43 (6.6%)	225 (34.8%)	348 (53.9%)	73 (11.3%)	230 (35.6%)	416 (64.4%)
	Girls	603 (80.9% ^g^)	132 (17.7%)	10 (1.4%)	332 (44.5%)	358 (48%)	55 (7.5%)	246 (33.1%)	429 (57.4%)	69 (9.5%)	324 (43.4% ^f^)	423 (56.6%)
Economic and educational level												
University degree ^d, e^												
	Completed by mothers	277 (79%)	65 (18.5%)	9 (2.5%)	154 (44.2%)	170 (48.6%)	25 (7.2%)	100 (29.6%)	208 (59.4%)	41 (11%)	102 (29.1% ^f^)	248 (70.9%)
	Not completed by mothers	710 (72.2%)	246 (25.1%)	27 (2.7%)	437 (44.3%)	480 (48.7%)	69 (7%)	353 (35.9% ^g^)	533 (54.3%)	97 (9.8%)	427 (43.4% ^f^)	558 (56.6%)
	Completed by fathers	290 (80.4%)	65 (18%)	6 (1.6%)	154 (42.7%)	180 (50.1%)	26 (7.2%)	95 (26.5%)	218 (60.9%)	45 (12.6%)	116 (32.2% ^f^)	244 (67.8%)
	Not completed by fathers	636 (72.3%)	217 (24.6)	27 (3.1%)	399 (45.3%)	423 (47.9%)	60 (6.8%)	331 (37.5% ^g^)	468 (53.1%)	82 (9.4%)	368 (41.7% ^f^)	514 (58.3%)
Standard of living ^c^												
	Good	348 (77.1%)	90 (19.9%)	14 (3%)	200 (44.2%)	215 (47.6%)	37 (8.2)	151 (33.5%)	258 (57.1%)	42 (9.4%)	155 (34.2% ^f^)	298 (65.8%)
	Poor	677 (72.4%)	233 (25%)	24 (2.6%)	414 (44.3%)	462 (49.4%)	59 (6.3%)	320 (34.3%)	513 (55%)	100 (10.7%)	395 (42.3% ^f^)	539 (57.7%)
School dental services												
	Schools with dental office	617 (80.4% ^h^)	135 (17.5%)	17 (2.1%)	347 (45.3%)	367 (47.8%)	53 (6.9%)	221 (28.8%)	481 (62.7% ^g^)	66 (8.5%)	270 (35.2% ^f^)	497 (64.8%)
	Schools without dental office	414 (66.6%)	188 (30.1%)	21 (3.3%)	269 (43%)	312 (49.8%)	45 (7.2%)	250 (40.2%)	296 (47.5%)	76 (12.3%)	284 (45.4% ^f^)	342 (54.6%)
Regions of Bucharest												
	Zone A (central area)	300 (75.6%)	92 (23.1%)	5 (1.3%)	208 (52.5%)	166 (41.9%)	22 (5.6%)	147 (37.2%)	192 (48.6%)	56 (14.2%)	131 (33.1%)	265 (66.9%)
	Zone B (middle area)	460 (72.3%)	157 (24.7%)	19 (3%)	269 (42.2%)	331 (51.8%)	38 (6%)	192 (30.2%)	392 (61.6%)	52 (8.2%)	258 (40.4%)	381 (59.6%)
	Zone C (peripheral area)	212 (74.1%)	64 (22.4%)	10 (3.5%)	103 (36%)	152 (53.2%)	31 (10.8%)	107 (37.4%)	147 (51.4%)	32 (11.2%)	141 (49.3%)	145 (50.7%)
	Zone D (outmost periphery)	59 (80.8%)	10 (13.7%)	4 (5.5%)	36 (49.3%)	30 (41.1%)	7 (9.6%)	25 (34.2%)	46 (63.1%)	2 (2.7%)	24 (33.3%)	48 (66.7%)
TOTAL		1031 (74.1%)	323 (23.2%)	38 (2.7%)	616 (44.2%)	679 (48.7%)	98 (7.1%)	471 (33.9%)	777 (55.9%)	142 (10.2%)	554 (39.8%)	839 (60.2%)

The letters indicate ^a^ 4 missing data, ^b^ 3 missing data, ^c^ 6 missing data, ^d^ 58 missing data (for mothers), ^e^ 151 missing data (for fathers), ^f^ *p* < 0.05, ^g^ *p* < 0.05 (different from both other answers), ^h^ *p* < 0.05 compared with one answer and *p* = 0.06 compared with another.

## Data Availability

The data presented in this study are available upon request from the corresponding author.
